# Estimation of daily sodium and potassium excretion from overnight urine of Japanese children and adolescents

**DOI:** 10.1186/s12199-020-00911-3

**Published:** 2020-11-27

**Authors:** Masayuki Okuda, Keiko Asakura, Satoshi Sasaki

**Affiliations:** 1 Graduate School of Sciences and Technology for Innovation, Yamaguchi University, 1-1-1 Minami-Kogushi, Ube, 755-8505 Japan; 2 Department of Social Medicine, Faculty of Medicine, Toho University, 5-21-16 Omori-Nishi, Ota-ku, Tokyo, 143-8540 Japan; 3 Department of Social and Preventive Epidemiology, School of Public Health, The University of Tokyo, 7-3-1 Hongo, Bunkyo-ku, Tokyo, 113-0033 Japan

**Keywords:** Adolescents, Children, Creatinine, Estimation, Excretion, Potassium, Sodium, Urine

## Abstract

**Background:**

Estimates of daily sodium (Na) and potassium (K) excretion were explicitly biased when using equations for adults. We aimed to develop equations to estimate them using overnight urine from Japanese children and adolescents.

**Methods:**

The subjects comprised 70 students aged 10.49–15.76 years: validation group, *n* = 34; and verification group, *n* = 36. Each subject performed two operations of overnight spot urine (*U*_m_) and 24-h urine (U_24_) sampling. Concentrations of Na, K, and creatinine (Cr) were measured, and anthropometrics were recorded. In the validation group, Na/Cr, and K/Cr (mEq L^−1^/mg dL^−1^) in 24-h urine were predicted from their correspondents in overnight urine. Daily Cr excretion (EstCr_24_; mg d^−1^) was estimated according to Mage’s method.

**Results:**

In validation, we formulated Na excretion (mg d^−1^) = 23 × exp (0.2085) × [(Na/CrU_m_ + 1)^1.0148^ − 1] × 1.078 × EstCr_24_/10; and K excretion (mg d^−1^) = 39 × exp (0.0315) × [(K/CrU_m_ + 1)^1.3165^ − 1] × 1.078 × EstCr_24_/10. For verification, we compared estimates with the measured 24-h Na excretion 3596 ± 1058 mg d^−1^, and K excretion 1743 ± 569 mg d^−1^. The mean biases and intraclass correlations (3, 1) were −131 mg d^−1^ and 0.60, respectively for Na excretion; and −152 mg d^−1^ and 0.55 for K excretion.

**Conclusion:**

We obtained validated equations to estimate daily Na and K excretion with accessible variables such as Na, K, and Cr concentrations of overnight urine, body height and weight, and age for children and adolescents. When using the obtained equations, caution should be paid to small but definite biases and measurement errors.

**Supplementary Information:**

The online version contains supplementary material available at 10.1186/s12199-020-00911-3.

## Background

High sodium (Na) intake is the leading dietary risk factor for death and disability-adjusted life years in East Asia and high-income Asia Pacific regions [1]. The World Health Organization (WHO) recommends reduced sodium intake for children to control blood pressure [2]. Potassium (K) supplementation decreases blood pressure in hypertensive patients with or without antihypertensive drugs, particularly in high sodium consumers [3]. Na and K have opposite effects on blood pressure.

Excess of sodium intake in Japanese is one of the public health challenges targeting children and adolescents as well as adults. Objectively measuring and understanding the salt and potassium consumption of the population is the first step in a public health intervention. However, estimates of Na and K excretion from spot urine of secondary school adolescents using equations for adults were explicitly biased: 0.6-1.4 g in absolute values for Na, and 35-628 mg for K [4]. In a study of other sample using another equation for adults, the salt intake of Japanese adolescents has been estimated from spot urine as 7.6 g/d [5], which is lower than the 10.0–10.6 g/d figures using 24-h urine [4]. Equations for adults had large biases when applying them for adolescents [4], and there were no other equations for youths.

Several equations to estimate salt intake from spot urine for adults have been proposed, and include regression methods [6, 7], and the Na/creatinine (Cr) ratio multiplied by daily Cr excretion [8, 9]. We have previously used regression models to explore the association with the daily excretion of Na and K in secondary school adolescents [4], which demonstrated that demographic variables such as age and weight were not determinants in contrast to the previously proposed equations [6–9], and the correlation coefficients of estimates were < 0.5 [4].

The aim of this study was to develop equations using the ratios of Na/Cr, and K/Cr according to Kawasaki and Tanaka methods [8, 9] to estimate daily urinary Na and K excretion using first-morning spot urine (overnight urine) in school-aged children and adolescents. We added data of primary school children to data of secondary school adolescents previously reported [4].

## Materials and methods

### Subjects

Volunteer subjects were recruited from 7th to 9th graders at 4 secondary schools (adolescents) in Suo-Oshima Town, Japan from 2014–2015, and 5th and 6th graders at 9 primary schools (children) in Suo-Oshima Town and Ube City, Japan from 2019–2020. The protocol has been described elsewhere [4, 10]. None of the subjects had hypertension, diabetes, heart disease, or kidney disease.

### Urine analysis

The subjects took a 10-ml sample of overnight urine (first-morning void), discarded the remaining overnight urine at the starting day, and collected subsequent 24-h urine until the first void the next morning; this operation was performed twice with an interval > 6 days. The subjects recorded the time of the first and last sampling, and the time if they mistakenly discarded. The urine samples were stored in a cool box until they were brought to the school. A collection period (t_clct_; min) was used to correct 24-h excretion. We measured a volume of 24-h urine (VoU; ml) and took another 10-ml sample from it. Four samples from each student were analyzed to measure the concentrations of Na (mEq L^−1^) and K (mEq L^−1^) using electrode methods, and Cr (mg dL^−1^) using enzyme test at the LSI Medience Corporation or the Yamaguchi Laboratory Co. Ltd.; the methods between laboratories were standardized periodically. Daily excretion of sodium and potassium (Na_24_ and K_24_, respectively; mg d^−1^) were determined in the following equations [4]:

1 $$ {Na}_{24}=23\times Na{U}_{24}\times VoU\times 1440/{t}_{clct}\div 1000 $$

2 $$ {K}_{24}=39\times K{U}_{24}\times VoU\times 1440/{t}_{clct}\div 1000 $$

where NaU_24_ (mEq L^−1^), and KU_24_ (mEq L^−1^) are sodium, and potassium concentrations in 24-h urine, respectively. Daily creatinine excretion (Cr_24_; mg d^−1^) was determined in the following equation:

3 $$ {Cr}_{24}= Cr{U}_{24}\times VoU\times 1440/{t}_{clct}\div 100 $$

where CrU_24_ (mg dL^−1^) is the concentration of creatinine in 24-h urine.

### Demographic data

Body height (Ht, cm) and weight (Wt, kg) were measured at school. The body mass index (BMI) was calculated as Wt (kg)/Ht (m)^2^, and obesity was defined using cut-offs of the International Obesity Task Force, which indicates a corresponding prevalence of 30 kg m^−2^ at 18 years old [11]. The standard BMI (stdBMI) was calculated based on the Japanese reference [12]. Ages (year) were calculated to two decimal places by dividing the difference between the birth date and the first sampling date by 365.25.

### Subject selection

Subjects with at least one of the following criteria were excluded from the analysis: (1) discarding void at least once, or (2) t_clct_ < 1200 min in either of the two operations. Subjects with two complete 24-h urine collections were randomly allocated into a validation group at a sampling rate of 0.5, and remaining subjects were allocated into a verification group, using the SAS SURVEYSELCT procedure (SAS Institute Japan Inc., Tokyo, Japan).

### Estimation

The Na/Cr or K/Cr in 24-h urine ([Na/Cr]U_24_ or [K/Cr]U_24_, respectively) were predicted using regression models, whereby the estimates were Est[Na/Cr]U_24_ or Est[K/Cr]U_24_, respectively. The predictors were mean overnight urine of each subject, [Na/Cr]U_m_, or [K/Cr]U_m_, and the outcomes were the mean of measured [Na/Cr]U_24_ or [K/Cr]U_24_. After each variable plus one was natural-log-transformed, the Passing-Bablock regression method with bootstrap estimation (R *mcr* package; CRAN R project) was used because this method can avoid regression dilution derived from measurement errors of predictors, and is robust to outliers [13].

4 $$ \ln \left( Est\left[ Na/ Cr\right]{U}_{24}+1\right)={a}_{Na}+{b}_{Na}\times \ln \left(\left[ Na/ Cr\right]{U}_{\mathrm{m}}+1\right) $$

5 $$ \ln \left( Est\left[K/ Cr\right]{U}_{24}+1\right)={a}_K+{b}_K\times \ln \left(\left[K/ Cr\right]{U}_{\mathrm{m}}+1\right) $$

where *a* is an intercept, and *b* is a slope. Daily Cr_24_ was estimated using Mage’s equation (EstCr_24_; Additional file 1). The ratio of measured Cr_24_ to EstCr_24_ was determined as a factor to correct estimates.

### Verification

In the verification group, the estimated daily excretion of sodium or potassium (EstNaU_24_ or EstKU_24_) as the mean of two operations in each subject was calculated followed by the methods of Kawasaki and Tanaka [8, 9].

6 $$ Est{Na}_{24}\propto Est\left[ Na/ Cr\right]{U}_{24}\times Est{Cr}_{24} $$

7 $$ {EstK}_{24}\propto Est\left[K/ Cr\right]{U}_{24}\times {EstCr}_{24} $$

Estimates from overnight urine were compared to the measured values in 24-h urine; in order to assess the agreement between these measures, a fixed model intraclass correlation ICC (3, 1) was calculated using the R *psych* package (CRAN R project), and Bland-Altman plots were depicted (R *BlandAltmanLeh* package).

### Other statistical methods

Continuous variables were expressed as mean ± standard deviation, and categorical variables were expressed as count (%). Spearman’s correlation coefficient was used to assess the associations between two variables. Units of “mEq L^−1^” and “mg dL^−1^” were used for concentrations as returned from the laboratories with SI units. Sodium intake (mEq) was expressed as a unit “mg” multiplied by 23, or equivalent salt (g) multiplied by 58.5/1000. Potassium (mEq) was expressed as a unit “mg” multiplied by 39.

## Results

### Subjects

Demographic characteristics and urinalysis for the subjects were presented in Tables 1, 2, and Additional file 2. Of 91 volunteer subjects, 21 subjects were excluded for the following reasons: 16 subjects lost urine at least once, and 5 subjects had urine collection < 1200 min. From the remaining 70 subjects, 34 were randomly allocated to the validation group, and 36 were allocated to the verification group.

**Table 1:** Demographic characteristics of the subjects (*n* = 70)

	Validation, *n* = 34	Verification, *n* = 36
Male, %	47.1	44.4
Primary school students, %	29.4	30.6
Age, year	13.41 ± 1.40	13.24 ± 1.44
(Minimum, maximum)	(10.78, 15.32)	(10.49, 15.76)
Body height (Ht), cm	155.6 ± 7.1	155.2 ± 9.7
Body weight (Wt), kg	50.6 ± 9.6	49.1 ± 12.4
Obesity, %	2.9	8.3

**Table 2:** Urinalysis of the validation group (*n* = 34)

	First operation	Second operation
24 h urine (U_24_)		
Collection periods (t_clct_), min	1367 ± 86	1379 ± 69
Volume (VoU), mL	981 ± 446	932 ± 406
NaU_24_, mEq L^−1^ (mmol L^−1^)	155.7 ± 49.5	166.6 ± 48.6
KU_24_, mEq L^−1^ (mmol/L^−1^)	44.14 ± 16.4	49.4 ± 20.5
CrU_24_, mg dL (× 10/113 mmol L^−1^)	108.0 ± 47.3	119.4 ± 47.8
[Na/Cr]U_24_, mEq L^−1^/mg dL^−1^	1.63 ± 0.65	1.56 ± 0.55
[K/Cr]U_24_, mEq L^−1^/mg dL^−1^	0.46 ± 0.26	0.43 ± 0.12
Na_24_, mg d^−1^	3346 ± 1082	3476 ± 1184
K_24_, mg d^−1^	1587 ± 483	1723 ± 664
Cr_24_, mg d^−1^	970 ± 313	1045 ± 311
Overnight urine (U_m_)		
NaU_m_, mEq L^−1^ (mmol L^−1^)	118.4 ± 47.7	119.1 ± 53.3
KU_m_, mEq L^−1^ (mmol L^−1^)	40.8 ± 16.3	35.0 ± 16.6
CrU_m_, mg dL^−1^ (× 10/113 mmol L^−1^)	152.5 ± 59.0	133.9 ± 57.5
[Na/Cr]U_m_, mEq L^−1^/mg dL^−1^	0.93 ± 0.60	1.00 ± 0.43
[K/Cr]U_m_, mEq L^−1^/mg dL^−1^	0.29 ± 0.12	0.28 ± 0.10

NaU, KU, CrU: sodium, potassium, and creatinine concentrations in urine, respectively; [Na/Cr]U, [K/Cr]U, [Na/K]U: ratios of sodium-to-creatinine, potassium-to-creatinine, and sodium-to-potassium concentrations in urine, respectively; U_24_, U_m_: 24-h urine, and overnight urine, respectively; Na_24_, K_24_, Cr_24_: measured daily urinary excretion of sodium, potassium, and creatinine, respectively

### Validation

EstCr_24_ were 940.7 ± 161.3 mg d^−1^, and its correlation coefficient with measured Cr_24_ (1007.3 ± 281.7 mg d^−1^; Table 2, and Additional file 2) was 0.59. Thereafter, we used EstCr_24_ multiplied by 1.078 (1007.3/940.7).

Regression slopes and intercepts to predict Na/Cr, and K/Cr in 24-h urine were obtained using the Passing-Bablock method (Table 3). Finally, based on these parameters, the estimation equations for Na_24_ and K_24_ are obtained:

8 $$ Est{Na}_{24}=23\times \exp (0.2085)\times \left\{{\left(\left[ Na/ Cr\right]{U}_m+1\right)}^{1.0148}-1\right\}\times 1.078\times Est{Cr}_{24}\div 10 $$

9 $$ {EstK}_{24}=39\times \exp (0.0315)\times \left\{{\left(\left[K/ Cr\right]{U}_m+1\right)}^{1.3165}-1\right\}\times 1.078\times {EstCr}_{24}\div 10 $$

**Table 3:** Regression models for Est[Na/Cr]U24, and Est[K/Cr]U24 in the validation group (*n* = 34)

	Slope (95% confidence limits)	Intercept (95% confidence limits)
ln(Na/Cr +1)	1.0148 (0.5177, 1.9943)	0.2085 (−0.4007, 0.5393)
ln(K/Cr +1)	1.3165 (0.8290, 3.0075)	0.0315 (−0.3682, 0.1501)

Est[Na/Cr]U24 and Est[K/Cr]U24: estimated ratios of sodium to creatinine and potassium to creatinine in 24-h urine

Passing-Bablok regressions were used for natural log-transformed variables. Units for Na (sodium), K (potassium), and Cr (creatinine) were mEq L^−1^, mEq L^−1^, mg dL^−1^, respectively

### Verification

**Fig. 1:**
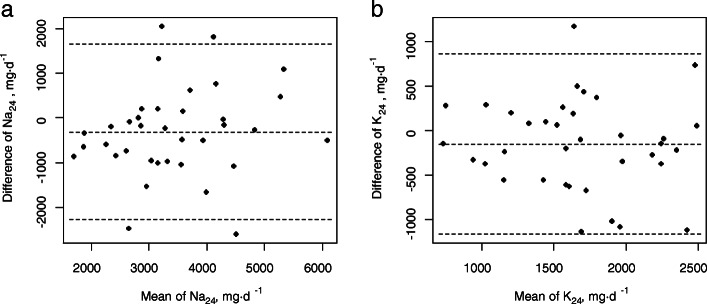
Bland-Altman plots of daily excretion of sodium (Na_24_) and potassium (K_24_) in the verification group. Horizontal axes indicate the mean of estimates (Est) and measurements, and vertical axes indicate the differences (estimates−measurements). Dashed lines indicate mean biases (mean ± 1.96 standard deviation): −309 (−2275, 1658) for EstNa_24_ (**a**); −152 (−1165, 861) for EstK_24_ (**b**). *n* = 36

The daily Na and K excretion in 24-h urine was estimated using the obtained equations in the verification group (Table 4, and Fig. 1). The mean biases of EstNa_24_ and EstK_24_ were less than 10% of the mean measured values, and the ICCs (3, 1) were 0.60 and 0.55, respectively.

**Table 4:** Verification (*n* = 36)

	24-h excretion (mean ± SD)	Estimation (mean ± SD)	ICC (3,1) (95% confidence limits)
Na_24_, mg d^−1^	3596 ± 1058	3288 ± 1210	0.61 (0.40, 0.76)
K_24_, mg d^−1^	1743 ± 569	1591 ± 520	0.55 (0.33, 0.72)

*ICC* intraclass correlation, *Na* sodium, *K* potassium, *SD* standard deviation

## Discussion

In this study, we formulated estimation equations for daily urinary Na and K excretion using overnight urine in Japanese children aged 10–15 years. Estimates of daily Na and K excretions showed 0.55–0.60 in ICCs (3, 1) as indices of criterion validity, which are capable of providing de-attenuate coefficients in future studies.

Tanaka’s equation was used in previous studies that estimated daily Na excretion in Japanese children [5, 14]; primary school children had Na excretion that was equivalent to 6.3–7.8 g d^−1^ salt. In the current study, using Tanaka’s equation, the estimate of daily Na excretion was equivalent to 7.3 g d^−1^ salt (Additional file 2); this estimate is lower than the measured value. Underestimation of Na excretion may be attributed to using Tanaka’s equation, but not to sample variation. Possible explanations for underestimation are as follows. First, Na excretion has diurnal variation [15], and Na and K concentration is lower during the night [15]; however, Tanaka et al. did not use overnight urine [9]. Second, we used chronological ages instead of fiscal ages; thus, when the birth date is unavailable, ages might have been integral numbers, which are younger than chronological ages. For example, the mean age of 7th graders is 11.5 (range, 11.0–11.9) years at the beginning of the fiscal year. Third, Tanaka’s equation was developed for adults aged 20–59 years, where age is used to estimate daily Cr excretion. EstCr_24_ was biased negatively in the primary school children more than in the secondary school adolescents (125 mg d^−1^ vs. 18 mg d^−1^; Additional file 2); thus, estimates may be more biased in younger children.

Body composition is different between Asians and Europids [16]. Whether Mage’s equation [16] is applicable to Japanese children and adolescents is currently unknown. Mage’s equations underestimated EstCr_24_ in the Japanese subjects of this study, and it is possible that daily Cr excretion in Japanese individuals is lower than that in Europids.

The Japanese Ministry of Health, Labour, and Welfare have proposed a tentative dietary goal for preventing life-style related diseases for these age groups: less than 6.0–7.0 g and more than 2200–2400 mg for Na and K intake, respectively [17]. Considering extra-urinary excretion in the 24-h values of the total subjects (Additional file 2), the subject aged 10.5–15.8 years had a salt intake of 9.9-10.5 g d^−1^, which are higher than the references, and had potassium intake of 2042–2279 mg d^−1^, which were lower.

There are several limitations of this study. First, the sample size was small, and as a result, the mean of measurements and estimates varied among sub-groups. We used a robust method, Passing-Bablock regression, to formulate the equations, and presented the extents of measurement errors. Second, the subjects’ resident areas were limited in Yamaguchi Prefecture. Equations can be applied to children and adolescents within the ranges of measured values. Third, if sex-specific equations were developed, more fitting equations could be generated; however, the slopes and intercepts were similar between sexes. Fourth, the 24-h collected urine did not involve the overnight urine; thus, the associations between predictors in the overnight urine and outcomes in the 24-h urine may be lower than that if spot urine was involved in the 24-h urine. Furthermore, the correlation coefficients of Na concentrations between overnight and 24-h urine in this study (0.37–0.49) were lower than those in the previous report (0.71) [18]. However, we used the mean of two operations, > 6 days apart, in order to estimate usual intake, which might partly compensate this weakness; the correlation coefficient was improved to 0.57.

## Conclusions

We obtained validated equations to estimate daily Na and K excretion with accessible variables such as Na, K, and Cr concentrations of overnight urine, body height and weight, and age for children and adolescents. Using the obtained equations, urine sampling at compulsory annual health checkups in school is useful to estimate salt and K intake without their additional burden unless urine sampling at another time. When estimating salt and K intake, caution should be paid to biases and measurement errors.

## Supplementary Information

**Additional file 1.** Mage’s equation for estimating daily creatinine excretion (DOCX 23 kb)

**Additional file 2.** Measured, and estimated excretion of creatinine, sodium, and potassium (DOCX 23 kb)

## Data Availability

The dataset used during the current study is available from the corresponding author on reasonable request.
