# Supplementary material for: Estimation of daily sodium and potassium excretion from overnight urine of Japanese children and adolescents
Source: Environ Health Prev Med. 2020 Nov 27;25:74. doi: 10.1186/s12199-020-00911-3 (PMC7697364; doi:10.1186/s12199-020-00911-3)
Supplement: Supplementary file 1 — Additional file 1. Mage’s equation for estimating daily creatinine excretion (DOCX 23 kb) [file 12199_2020_911_MOESM1_ESM.docx]

**Additional file1.docx Mage’s equation for estimating daily creatinine excretion [1]**

For estimation of daily urinary creatinine excretion (EstCr24; mg∙d^-1^), age (years), Ht: Body height (cm), BMI: Body mass index (kg∙m^-2^), and stdBMI: BMI standard for sex, age, and height (kg∙m^-2^) were used as following equations:

1. If BMI > corresponding to 30 kgm^-2^ at 18 years old

- Males with body height > 168 cm

| $Est{Cr}_{24}=1.085\times Ht\times\left[ 6.265+0.2550\times\left( Ht-168 \right) \right]\times$ $(1.10-0.0128\times BMI)/(1.10-0.0128\times stdBMI)$*.* | (S1) |
| --- | --- |

- Males with body height ≤ 168 cm

| $Est{Cr}_{24}=1.085\times Ht\times\left[ 6.265+0.0564\times\left( Ht-168 \right) \right]\times$ $(1.10-0.0128\times BMI)/(1.10-0.0128\times stdBMI)$*.* | (S2) |
| --- | --- |

- Female

| $Est{Cr}_{24}=1.085\times Ht\times2.045\times exp\left[ 0.01552\times\left( Ht-90 \right) \right]\times$ $\left( 1.07-0.0148\times BMI \right)/\left( 1.07-0.0148\times stdBMI \right)$. | (S3) |
| --- | --- |

1. If BMI ≤ corresponding to 30 kgm^-2^ at 18 years old

- Males with body height > 168 cm

| $Est{Cr}_{24}=1.085\times Ht\times\left[ 6.265+0.2550\times\left( Ht-168 \right) \right]\times$ $\left\{ Wt/[14+1.433\left( age-3 \right)+0.22\left( age-3 \right)^{2}-0.00533\left( age-3 \right)^{3}] \right\}^{0.5}$. | (S4) |
| --- | --- |

- Males with body height ≤ 168 cm

| $Est{Cr}_{24}=1.085\times Ht\times\left[ 6.265+0.0564\times\left( Ht-168 \right) \right]\times$ $\left\{ Wt/[14+1.433\left( age-3 \right)+0.22\left( age-3 \right)^{2}-0.00533\left( age-3 \right)^{3}] \right\}^{0.5}.$. | (S5) |
| --- | --- |

- Females

| $Est{Cr}_{24}=1.085\times Ht\times2.045\times exp\left[ 0.01552\times\left( Ht-90 \right) \right]\times$ $\left\{ Wt/[14+0.40\left( age-3 \right)+0.52\left( age-3 \right)^{2}-0.024\left( age-3 \right)^{3}] \right\}^{0.5}$. | (S6) |
| --- | --- |

1. Mage DT, Allen RH, Kodali A. Creatinine corrections for estimating children's and adult's pesticide intake doses in equilibrium with urinary pesticide and creatinine concentrations. Journal of exposure science & environmental epidemiology. 2008;18:360–8. doi:10.1038/sj.jes.7500614.
