# Supplementary material for: Estimation of daily sodium and potassium excretion from overnight urine of Japanese children and adolescents
Source: Environ Health Prev Med. 2020 Nov 27;25:74. doi: 10.1186/s12199-020-00911-3 (PMC7697364; doi:10.1186/s12199-020-00911-3)
Supplement: Supplementary file 2 — Additional file 2. Measured, and estimated excretion of creatinine, sodium, and potassium (DOCX 23 kb) [file 12199_2020_911_MOESM2_ESM.docx]

**Additional file 2.docx Measured and estimated excretion of creatinine, sodium, and potassium**

|  | **Total, *n* = 70** | **Children, *n* = 21** | **Adolescents, *n* = 49** |
| --- | --- | --- | --- |
| **Cr_24_, mg∙d^-1^** | **1004 ± 259** | **916 ± 310** | **1041 ± 228** |
| **Na_24_, mg∙d^-1^** | **3506 ± 1020** | **3363 ± 1085** | **3568 ± 997** |
| *Na_24_ excretion, salt-eq. g∙d^-1^* | *8.9 ± 2.6* | *8.5 ± 2.8* | *9.1 ± 2.5* |
| *Na_24_ Intake, salt-eq. g∙d^-1^* | *10.4 ± 3.0* | *9.9 ± 3.2* | *10.5 ± 2.9* |
| **K_24_, mg∙d^-1^** | **1700 ± 534** | **1572 ± 568** | **1755 ± 516** |
| *K_24_ intake, mg∙d^-1^* | *2208 ± 694* | *2042 ± 738* | *2279 ± 670* |
| **EstCr_24_mage, mg∙d^-1^** | **931 ± 195** | **829 ± 169** | **975 ± 190** |
| **EstCr_24_tanaka, mg∙d^-1^** | **979 ± 270** | **790 ± 238** | **1060 ± 242** |
| **EstNa_24_tanaka, mg∙d^-1^** | **2883 ± 530** | **2729 ± 433** | **2948 ± 558** |
| *Na_24_ excretion, salt-eq. g∙d^-1^* | *7.3 ± 1.3* | *6.9 ± 1.1* | *7.5 ± 1.4* |
| *Na_24_ Intake, salt-eq. g∙d^-1^* | *8.5 ± 1.6* | *8.1 ± 1.3* | *8.7 ± 1.6* |
| **EstK_24_tanaka, mg∙d^-1^** | **1194 ± 188** | **1069 ± 150** | **1247 ± 178** |
| *K_24_ intake, mg∙d^-1^* | *1550 ± 244* | *1389 ± 194* | *1619 ± 232* |
| **EstNa_24_, mg∙d^-1^** | **3303 ± 1303** | **3154 ± 936** | **3276 ± 1367** |
| *Na_24_ excretion, salt-eq. g∙d^-1^* | *8.4 ± 3.3* | *8.0 ± 2.4* | *8.3 ± 3.5* |
| *Na_24_ Intake, salt-eq. g∙d^-1^* | *9.8 ± 3.8* | *9.3 ± 2.8* | *9.7 ± 4.0* |
| **EstK_24_, mg∙d^-1^** | **1641 ± 514** | **1412 ± 417** | **1740 ± 524** |
| *K_24_ intake, mg∙d^-1^* | *2132 ± 667* | *1834 ± 541* | *2259 ± 680* |

Cr: creatinine; Na: sodium; K: potassium;
Unit ‘salt-eq. g’: salt equivalent gram was calculated Na (mg) multiplied by 2.54/1000;
Intake of Na and K was estimated from excretion of Na and K divided by 0.86 and 0.77, respectively according to Holbrook’s report [1];
Prefix “Est” indicates estimated values. Cr was estimated from over-night urine samples using Mage‛s (postfix ‘mage’) [2], and Tanaka‛s methods (postfix “tanaka”) [3]. Na and K were estimated using Tanaka‛s method [3], and equations obtained in this study (without postfix).

1. Holbrook JT, Patterson KY, Bodner JE, Douglas LW, Veillon C, Kelsay JL, et al. Sodium and potassium intake and balance in adults consuming self-selected diets. Am J Clin Nutr. 1984;40:786–93. doi:10.1093/ajcn/40.4.786.
2. Mage DT, Allen RH, Kodali A. Creatinine corrections for estimating children‛s and adult‛s pesticide intake doses in equilibrium with urinary pesticide and creatinine concentrations. J Expo Anal Environ Epidemiol. 2008;18:360–8. doi:10.1038/sj.jes.7500614.
3. Tanaka T, Okamura T, Miura K, Kadowaki T, Ueshima H, Nakagawa H, et al. A simple method to estimate populational 24-h urinary sodium and potassium excretion using a casual urine specimen. J Hum Hypertens. 2002;16:97–103. doi:10.1038/sj/jhh/1001307.
